# Rejection, acceptance and the spectrum between: understanding male attitudes and experiences towards conflict-related sexual violence in eastern Democratic Republic of Congo

**DOI:** 10.1186/s12905-017-0479-7

**Published:** 2017-12-08

**Authors:** Jocelyn Kelly, Katherine Albutt, Justin Kabanga, Kimberley Anderson, Michael VanRooyen

**Affiliations:** 1 Harvard Humanitarian Initiative, Harvard University, Cambridge, USA; 2 Department of General Surgery, Massachusetts General Hospital, Boston, USA; 3 Program in Global Surgery and Social Change, Harvard University, Cambridge, USA; 4 Centre D’Assistance Médico Psychosociale, South Kivu, Democratic Republic of the Congo; 5 Psychotraumacentrum Zuid Nederland, Reinier van Arkel Groep, Bethaniestraat 10, 5211 LJ ’s-Hertogenbosch, Netherlands

**Keywords:** Sexual violence, Stigmatisation, DRC, Focus group, Male perspective

## Abstract

**Background:**

Female survivors of sexual violence in conflict experience not only physical and psychological sequelae from the event itself, but often many negative social outcomes, such as rejection and ostracisation from their families and community. Male relatives – whether husbands, fathers, brothers – play a key role in determining how the family and community respond to a survivor of sexual violence. Understanding these perspectives could help improve services for survivors of sexual violence, as well as their families and communities.

**Methods:**

This study draws on qualitative data gathered from focus groups of 68 men in the eastern region of Democratic Republic of Congo. Men were asked about their experiences as relatives of women who had experienced sexual violence.

**Results:**

Two dominant themes arose throughout the focus groups: factors driving rejection and pathways to acceptance. Factors driving rejection included: fear of sexually transmitted infections, social stigma directed toward the husbands themselves, and an understanding of marriage and fidelity that is incompatible with rape. Men also touched on their own trauma, including struggling with witnessing a rape that took place in public, or caring for a survivor with a child from rape. They noted that the economic burden of medical treatment for survivors was a salient factor in the decision to reject. Pathways to acceptance included factors such as the love of their spouse or relative, survivors’ potential to give continued financial contribution to the family, the need to keep the family together to care for children in the home, and pressure from people of importance in the community.

**Conclusion:**

This study provides unique insight into how male relatives respond to close family members who have experienced sexual violence. This is particularly critical since the reaction of a male relative after rape can be the most pivotal factor in promoting or impeding recovery for a survivor. These results emphasise the importance of services that focus not only on the survivor of violence herself, but also on key family members that can ideally help support her recovery.

**Electronic supplementary material:**

The online version of this article (10.1186/s12905-017-0479-7) contains supplementary material, which is available to authorized users.

## Background

Congolese men and women have used the word ‘destruction’ to describe consequences of sexual violence^1^ in the conflict-affected eastern region of the Democratic Republic of Congo (DRC) [1]. This descriptor is used to express the wide-ranging and long-term consequences of rape on individuals, families and communities. Not only is rape often physically and psychologically devastating, but it also brings the fear of sexually transmitted infections, the risk that a survivor will no longer be accepted by members of their own families, and the breakdown of wider communal and familial relations. One recent ethnographic study explored the effect of sexual violence on social structures and masculinity with 231 men and women in eastern DRC [2]. The dominant ideals of masculinity that were described by respondents related to a man’s ability to provide for and protect his wife and children. However, these masculine ideals did not always reflect the reality of men’s lives. The study described how strong cultural and ethnic traditions regarding sexuality, gender roles and marriage in Congolese society may govern how survivors of sexual violence – and their families – are treated by the wider community. Sexual violence is a perceived assault on a family’s identity and challenges the highly proscribed customs related to a man’s role as protector and provider for the family [3]. Sexual violence perpetrated against a female relative has been associated with feelings of shame, inadequacy and loss of social status for both women and the men in their family. Social taboos dictate that victims of sexual violence be seen as unclean, spoiled or unfaithful, leading to protracted social isolation and stigmatisation [4, 5]. Injuries and trauma from sexual violence may also raise the concern that women may not be able to work, perform household chores or care for children; effectively diminishing their perceived ‘worth’ [6]. This accumulation of culturally bound expectations and beliefs for both men and women regarding masculinity, ‘womanhood’ and what constitutes a successful marriage, inform family and community responses to sexual violence. Depression and other psychological trauma may also create further barriers to intimacy with family and friends, may cause decreased school and church attendance, and diminish participation in wider community life [7] for women. Survivors of sexual violence in eastern DRC have stated that the stigma and shame they feel after rape can be more traumatic than the attack itself, since it may lead to isolation and, in many cases, women being disowned from their families [1]. The same study highlights the critical role male relatives play in influencing how a survivor is treated after a violent incident. They can either promote acceptance and healing, or be the deciding influence for rejection. Male relatives, as traditional providers, may also be critical factor in determining whether a survivor has the support and funds to seek care.

The highly brutal and public forms of rape perpetrated in DRC further exacerbate the stigma associated with this type of violence. In one report, Bartels and colleagues [8] documented more than half of women seeking care at a hospital in Sud-Kivu province of DRC as having been attacked in their own homes and in the presence of family members. Familial dynamics are often deeply disturbed by the traumatic stress reactions of family members forced to witness and participate in acts of sexual violence [9]. Although varying estimates exist regarding the percentage of sexual violence perpetrated by armed individuals, of the 11,769 cases of sexual and gender-based violence (SGBV) documented in a UN security report from January to September 2014 in eastern regions of DRC [10], 39% were reportedly committed by armed individuals. Further, 83 % of 255 survivors surveyed in a report by the Harvard Humanitarian Initiative [11] testified to having experienced rape by a uniformed perpetrator. In other areas, sexual violence perpetrated by armed actors can further exacerbate the stigma associated with rape, since victims may be believed to be colluding with the enemy – these women may be labelled “wives” of the rebels [12].

Pressures such as these can lead survivors to be abandoned by their spouses, families and communities, or be forced to leave their own homes [13]. Equally, these pressures can permeate society and extend to family members, especially husbands, who may also be targeted with stigmatisation. Yet, there is currently a paucity of research on the perceptions of the family and community towards sexual violence survivors.

A recent qualitative study of males in eastern DRC, [14] highlighted a consensus that a man would have to reject his wife following rape, as a means of upholding his honour. While in individual interviews some men were able to show compassion towards female sexual violence survivors, in general there was an overriding scepticism toward concepts of gender equality and messages about the importance of supporting survivors of sexual violence. Female participants in the same study echoed these results – noting that gender norms are highly unequal and rejection of survivors is common. In another study, [15] thirteen female survivors of sexual violence and three of their husbands were interviewed in an attempt to explore the shame directed at male relatives of survivors. Many of the study participants described how they were forced out of their homes or abandoned by their partners following sexual violence. When asked about these behaviours, one man described the “misunderstandings [that] are created between a couple, members of the family and people of their community that lead to the decision to reject a woman from the family” (page 744). Another husband who was a witness to his wife’s rape said that he is haunted by images of that night, and that accepting her, and the losses they have faced, was becoming increasingly difficult. The authors of this study reiterate the concern that some societies fail to distinguish between whether or not a woman gave consent to sexual contact (including rape), and many still believe she “wanted what happened” (page 746). Conversely, women in another study note that acceptance by a husband or other family member can serve as a transformational experience. Being supported, cared for, and encouraged to seek medical and psychological support has been described as survivors as one of the most important factors in healing and rebuilding their lives [1]. These findings highlight the marital complexity when a female is afflicted by sexual violence, but require further investigation.

The sensitive questions surrounding spousal acceptance, rejection and the spectrum in between, are therefore vital for understanding how to improve long-term healing for survivors. The objectives of this study are to explore the most salient influences on a man’s decision navigate complex decision points related to rejecting or accepting a female relative who has survived sexual violence by means of focus group discussions, to build on a small but growing evidence base that explores male relatives perceptions of SGBV, and to enhance the understanding of the complex spousal and community dynamics for survivors of SGBV in conflict-affected communities across eastern DRC.

## Methods

### Design and setting

Eight focus groups consisting of between six and eleven participants were conducted with men between December 2011 and January 2012 in the local communities of Kalehe, Katana, and Kabare, surrounding Bukavu, Sud-Kivu. Kalehe and Katana sites were selected because of high reported rates of sexual violence reported to the partner organisation at the time of the study. Kabare was selected because this area had limited access to services related to treating sexual violence at the time of the study according to the local partner organisation, and could provide insight into attitudes in an area without awareness raising related to stigma. For this reason, participants at Kabare were recruited through a community-based organisation not involved in direct service provision related to SGBV. Recruitment was conducted at a local non-governmental organisation (NGO), Centre d’Assistance Médico Psychosociale (CAMPS), and at Panzi Hospital in Bukavu, Sud-Kivu, which has considerable experience in treating survivors of sexual violence.

Topic guides and prompts were created for group facilitators to learn more about the local communities of these men and their beliefs regarding sexual violence of female relatives (see Additional file 1). Before each focus group, a consent script was presented and questions about the study were answered. The consent script emphasised the voluntary nature of participation and the fact that participants could discontinue the participation at any time. All focus groups were conducted in a private setting and participants underwent a thorough introduction to the session that emphasised the importance of keeping the topics discussed within the research confidential. Participants in the study provided verbal informed consent before taking part in any research activities. The interview was tested and refined with male social workers from CAMPS.

This study was approved by the internal review board of the Harvard School of Public Health and a Congolese Community Advisory Board of subject matter experts. All members of the research team underwent training in ethical research practices before carrying out the study.

### Coding and analysis

Two members of the research team first separately reviewed the focus group transcripts and coded the data based on initial salient patterns that emerged. Key themes were collaboratively defined, generating a codebook by which to begin the iterative process of ensuring data was adequately reflected. This process allowed for the identification of key themes and overarching narratives. Coding was conducted in NVivo V9.0 (QSR International, Cambridge, MA).

## Results

Sixty-eight men participated across the three sites, in focus groups consisting of between six and eleven participants. The average size of each discussion was 8 people with an average age of 52 years (range 28–88). Discussions were conducted in Swahili and were mediated by a Congolese male psychosocial counsellor with experience facilitating group discussions. Each session lasted between 90 and 120 min.

Findings from this study highlight the complexity of factors affecting a man’s decision to reject or accept a female relative who has survived sexual violence, and the degree of individual variability. While some factors were described as acting as a greater driving force to explain rejection than others, the diversity of responses suggests no single factor is identified as being holistically explanatory.

### Factors driving rejection

Three key themes arose consistently as principal drivers of rejection: a fear of sexually transmitted infections (STIs), negative attention directed at the husbands of survivors, and an understanding of fidelity and marriage that is incompatible with rape. Other factors arose less frequently, but were nonetheless pertinent to the discussion.

#### Fear of sexually transmitted infections

*“If my wife has been raped, I will be scared to sleep with her; I will be scared to death (Kalehe).”*

Each focus group had active and extensive discussions related to sexually transmitted infections (STI). There was a widespread perception that a woman who had been raped was inevitably infected with an STI, with particular emphasis on the fact that she might be infected with HIV. There was a strong focus in the discussions that if a man let a rape victim^2^ back into their home, he too would be at high risk of infection and even death. The fear of ‘contamination’ as influencing the rejection of survivors was widely perceived. As one participant explained of a rape victim:

*“Her husband does not easily accept to live with this woman because she has been raped – from fear. Fear she has HIV, or other opportunistic diseases like AIDS (Kabare).”*

Men described how the anxiety surrounding STIs was so overwhelming that, for the most part, rejection was seen as the only choice. As one focus group participant said: “If a girl or woman has HIV, then there is no way to accept her.” Equally, since sex is viewed as a central component of marriage, to abstain from this through fear of infection was described as unrealistic, and thus infection was likely inevitable:

*“We have agreed to live with them. For those with AIDS, we are also affected by the disease. Life will go on and we will die together. It is done. We are living with them, and we will die with them. That is the end of the story (Kalehe).”*

Some men noted that armed perpetrators of rape had the explicit purpose of infecting women and their families with disease as a way of destabilising Congolese society. One focus group member framed this intent to infect as a way of harming entire communities: “Sexual violence is a serious issue, how? Whoever started it wants to kill us all.” Some explained the belief that this intent to damage Congolese society was linked to armed combatants who arrived after the Rwandan genocide, “We have been affected because of the Rwandan occupation, the Rwandans brought in lots of diseases including AIDS.” Militarised rape in particular was seen by respondents to be a driving factor in rejection of female survivors of sexual violence.

#### Stigma against men

*"After rape, one loses self-esteem and respect.… people are laughing at you. That is when one lives in isolation and loneliness (Kalehe)."*

Focus group participants acknowledged and discussed the extent to which they have witnessed the stigmatising and ostracising of women who have been raped, in their local communities. Men also shared that if someone rapes their wife, the husband will likely become a direct target of humiliation and shame. Certain participants stated that communities’ isolate men whose wives have been raped. As one man described, “People stay away from me, and don’t want me to start a conversation with them.” Other forms of public humiliation were through gossip and ridicule, and the feeling that the opinions of the husband of a rape survivor were no longer listened to or respected. Many men felt their status in the community diminished, noting that people discredited their masculinity and labelled them a “useless person”, “not a real man”, “not normal” and, in some cases, challenged his Congolese identity by associating him with rebel forces.

*“There is a church at Kalambo where all the victims of sexual violence are rejected. The husband who is part of this church, when he goes there one tells him that ‘If you continue to be with the woman who was taken by the Rwandans and who became Rwandan Hutu at the same time (in becoming their woman), we will exclude you also and no longer be a part of the church.’ The husband also sees that he cannot…remain in the church. He will seek a healthy woman. (Kambamba)”.*

In the eyes of the community, the men felt they had lost their social status: “The man is at a loss because people in the community doubt his power. He failed his responsibility as a man”.

A man’s closest relations have a particularly strong influence over his decision to reject or accept a survivor of violence. Participants gave examples of relatives who also threatened to exclude them, or cut off their social ties. This led men to make painful decisions about how to respond after rape, as one respondent stated,

*“When you sit down and think about being dumped by your family because of your wife, you decide to leave your wife and keep the family ties intact (Kalehe).”*

#### Definitions of marriage

*“A woman belongs to just one man. This is our custom (Kabere).”*

In five of the eight focus groups, men discussed the way sexual violence disrupts the core tenants of marriage as they understand it, particularly through violating a sense of fidelity. Participants described how marriage was defined by a man having the sole right to sexual contact with his wife. Rape fundamentally violates this definition, leading a man to end a marriage he now sees as void. As one participant said, “If she has been with another man, I cannot be with her.” In some instances, the core definition of marriage rested on the idea of exclusivity, as one respondent noted, it is “exclusive sexual contact between a man and his wife.” Therefore, regardless of whether the sexual contact was voluntary, the act of having sexual contact with another man nullified the marriage. Another said, “Sharing a woman with another man is the problem. She is not food to share.”

Moreover, participants in all eight focus group discussions came to overwhelming consensus that sexual violence is non-consensual. Despite this, the nature of the discussions around SGBV showed changeable perceptions of whether women were ‘at fault’ for being raped. For some men, if a woman could prove she resisted the rape, he was convinced of her blamelessness, and therefore would be more likely to accept her. For others, if a survivor did not disclose her rape immediately (a phenomenon which is not uncommon given the stigma and negative consequences), this was proof of deceit and a cause for rejection. If men hear about the incident by word of mouth on the street, they would become more inclined to reject her:

*“The problem is because the man feels betrayed and thinks his wife had sex on purpose and hid it. That means she cheated on him. She sent you a message that she doesn’t care about you (Kalehe).”*

Focus group discussants explained that if a husband could be sure of his wife’s commitment to fidelity, he would be more likely to accept her. As one man remembered “how she behaved in our house previously, I will know that she was not a cheater, and keep her with me.”

For some men, understanding that the attack was “not her fault” could facilitate acceptance. As one participant said, “The victim was tied up, and everything else happened, how could you reject her?” Another man, speaking about reasons to accept survivors, answered: “First, she is forced into the drama. It was not her intention.” Another asserted:

*“Only an idiot husband tells her, ‘get out of my face because I don’t understand what you have done to me,’ even though the wife is telling him that the act was unintentional (Katana, 1).”*

Beyond marriage, the respective roles of men and women were discussed in great depth. A woman’s social “value” was shown to be closely associated with her reproductive exclusivity and ability to prove her children belonged to her husband. Women who are unable to fulfil their marital obligations to their husbands – because of trauma, infertility due to rape, or because of the real or perceived threat of STIs including HIV – were perceived as “damaged” and therefore poor partners. Some men described survivors as the “wives” of their rapists, sealing their fate as “outsiders” in the community:

*“As head of the family, I have two daughters who were taken by the rebels and brought into the bush. And the boys to whom they were previously betrothed refused to continue the relationship with them in saying that those who became women/wives of the Interahamwe can no longer be our wives. They were pirated. (Katana)”.*

### Other influences for rejection

#### Economic factors

*“You reject her because you don’t have enough money to get medical care for her (Katana)*”.

The economic situation of a household can play a central role in rejecting survivors of sexual violence. Often families lack the means to care for women in need of treatment considering the already impoverished state of households in this area of eastern DRC, and as one man explained:

*“You add the new needs and expenses due to disease from rape, and the man cannot afford this so he rejects the woman (Katana)”.*

The militarised use of sexual violence in DRC means that the act of rape is commonly associated with other atrocities, including the murder of a family member, theft, torture, beating, and burning of houses. In five of the eight focus groups, men described how rape is often accompanied by catastrophic economic losses. A man from Katana explained:

*“The reason that a man chases away his wife is the lack of financial resources, because once this happens to the woman, one cannot have the financial means to care for her and people advise the man to send away the raped woman because he cannot support the cost of medical care. The man will eventually reject her….”*

Often survivors are unable to work due to health issues resulting from the attack, compounding the family’s financial loss. One focus group participant described:

*“It is the women who bring in more money at home, because if she does not go out, one has difficulties living. These days it is women [rather than men] who are doing better by running by here and by there. As is my case, since my wife had this problem, she never leaves the house and survival has become difficult (Katana).”*

Respondents noted that the inability to work was due to both physical and psychological problems. Some men, though not many, discussed how the emotional trauma survivors experienced from stigmatising community members meant they no longer contributed towards the household income. One man from Katana said:

*“My wife was raped by the Interahamwe, since then she has not been well with other women, these other women no longer want her near them, if she sits on a chair, they will chase her saying that they do not want to be contaminated by the chair, she could only lock herself at home and that is how she lives. The plot of land that we had, I sold it to care for her problems of the lower abdomen about which she is always complaining, and she has not conceived since.”*

#### Men’s descriptions of their own trauma

*“The men are traumatised. You will cry inside. There is a need for a mechanism to de-traumatise men after rape [of their relative] (Kabare).”*

Respondents from all eight focus groups recognised how rape perpetrated against their wives had negative emotional and psychological effects on themselves, which in some cases lead to rejection. These emotional consequences ranged from anger and hostility, to sadness, fear, and pain. One man said,

*“The husband leaves for a new partner because he does not want to go back to the same wife where he feels pain*. *No matter what you do, the pain won’t dissipate (Kalehe).”*

In all focus groups, men described experiencing deep psychological distress as a result of sexual violence against a relative. Symptoms of this traumatic event included persistent and intrusive memories of the rape, anxiety, fear, and depression. However, only a few participants directly linked mental health problems with the decision to reject. Instead, participants often noted that more pressing social pressures – such as a sense of public shame and financial considerations – as the direct cause for rejection.

#### Public rape

*“When family members have witnessed the rape of their daughters… This act is equated to a deadly attack (Bukavu).”*

A number of focus group respondents described how they were forced to participate in sexual violence against a family member. This meant in some cases men were able to see that women protested, that it was “not their fault.” In other instances, however, respondents noted that the public nature of the attack meant that their “shame” was more widely known in the community. Often, rape in the presence of one’s family was perceived as an attack on the society as a whole, which resulted in a collective feeling of shame. A man from Sange explained,

*“A rape committed against a woman or girl in the presence of other family members is not perceived in the same way as rape without their knowledge. When this is done before members of the family, it affects everyone a lot, we seek to know more about how this happened. Whereas rape committed against a woman/girl in secret, we do not care to find out how it happened or how to prevent it.”*

Another man from Katana noted, “The family can watch the rape of their daughter or mother and this may push them to say that it should not be known outside because it is shameful for the whole family if it were to be divulged.” This was not, however, a universal response.

#### Children born of rape

*“…It isn’t as easy for the husband to accept the child he knows is not his (Bukavu).”*

Respondents described how women who have a pregnancy or child born from rape increases stigmatisation directed against survivors and their families; children born as a result of rape were seen as “reminders” of the attack and the accompanying traumatic events. Respondents explained that community members sometimes believe that the child will embody the worst qualities of their fathers, and women who give birth after rape are accused of raising a “replica” of the rapist. Mothers of these children are seen as sympathisers or “wives” of the men who attacked them.

### Pathways to acceptance

Men described myriad reasons why they might reject a survivor after rape. However, results from this study also highlighted several factors that supported the decision to accept survivors. These include love, financial contributions and children in the home, but required more prompts from group facilitators and were not as spontaneous as factors driving rejection.

#### Love and affection

*“As I was in deep love with her, my decision came easily to keep her (Kalehe).”*

Male respondents stated that sincere affection and love for their wives make men more likely to accept events that affect their marriage:

*“First of all, this is due to affection. There is a certain affection for her.… I am afflicted, but I have affection for her despite everything (Kabare).”*

Despite this, acceptance after rape is not a panacea for the many problems a couple might face after a rape. Many participants noted that they still experienced trauma, negative feelings and “heartache” at the recollection of the attack, “Because he has bad thoughts… Even if you logically want to stay with her, bad thoughts make it so that you don't want to be with her.” These negative emotional effects can be so intense that they negate feelings of affection in a previously healthy relationship.

*“Even if you love your wife, if you see her be raped, the love vanishes (Katana).”*

The findings highlight how feelings of affection can encourage acceptance of a survivor after rape, although negative feelings and trauma from the attack may continue to affect the family.

#### Women’s financial contributions to the household

While being seen a ‘drain’ on household savings can mean a survivor is expelled from the home, contributing to the family income is a powerful motivation for acceptance. Respondents explained how, if a survivor brings money into the home, most other negative reactions after rape are overlooked.

Furthermore, the ways in which people in the community react to survivors who contribute financially to their families are complex. In some instances, a woman’s ability to earn money was seen as a clear reason to keep her in the home. As one man said:

*“To the husband of the raped woman, several community members will come and tell him to profit from the income generated by the wife. And thus, the wealth or money of the raped woman becomes her protection at home (Sange).”*

However, in most cases, a survivor’s ability to earn income still did not negate negative social reactions. Both she and her family would still face stigma from the wider community, however the financial gain could serve as an incentive to remain together as a family. Another man from Sange said:

*“When a woman has managed to generate money or wealth, we have a tendency to accept her despite the rape because of her means. This is to say that we close our eyes to the criticism, especially the disgraces that friends issue in terms of arguing for rejection. Here the husband or family who had the intention of rejecting the raped woman finds themselves seduced by wealth or money of the victim.”*

#### Children in the home

*“A man worries about who will educate and care for his children without the woman, so he does not reject her (Kabare).”*

Having children in the home, and particularly young children who require dedicated care, can make it more likely that a survivor remains in her home after rape. Respondents stated that if a husband rejects his wife, his children might not be properly cared for: “A man cannot care for the children and the house like his wife can (Katana).”

However, survivors accepted only to care for children may face significant neglect in the family, in particular she may no longer be treated as a true wife. Focus group participants described how, if a man accepts a woman solely because of her capacity as a mother, she might not be accepted as a sexual partner. Thus, her role is no longer as a wife and partner but rather as a caretaker of the children. As one respondent explained:

*“The husband is destabilised too because he is uncertain between keeping his first wife to take care of his children, or marrying a new partner for his sexual desire (Kalehe).”*

*“[Y]our wife has been raped. … Get rid of her and get another woman. But you can keep her aside and keep helping her because she has helped you to give birth to your children. Look for someone suitable for you now. The previous one will still be in the picture, but the new one is the one to sleep with (Kalehe).”*

#### Pressure from people of influence

*“It is probable that he has gotten strength from people who encourage him to keep his wife (Kalehe).”*

While there were many actors who pressured men to reject survivors of violence, far fewer individuals were seen as promoting unity and kindness. In one group, when men were asked who promoted acceptance, the participants responded with laughter and incredulity, saying that “less than 1 %” of community members would encourage acceptance. Despite this strongly sceptical response, some participants did identify champions in some communities who tried to promote understanding.

In some cases, relatives and trusted friends could impart “good advice” encouraging family unity. One respondent even described how the survivor herself could turn to people of influence to make a case for acceptance:

*“His wife will go to [his neighbours and his brothers] and ask them to convince her husband not to reject her (Katana).”*

#### Religion

Men struggling to reconcile their feelings related to a survivor of violence cited personal faith and religious leaders as key supports. Men explained that certain churches and communities might actively promote the isolation of survivors. However, the majority stated that faith communities promoted understanding and tolerance. One man from Katana said:

*“The clarification that we have about raped women is that it is the churches who help in the advice they give to raped women, giving them comfort, encouraging them to not leave their home and to not think that it is the end of the world because of what happened to them.”*

#### Partial acceptance

The factors described above promote acceptance. However, they often do not result in a woman fully reintegrating into her household and marriage, because underlying trauma and associated feelings of stigma and shame remain unaddressed. Participants described how, even if a family remains intact, there is often still significant emotional distress and tension in both the household and community. Feelings of fear, anxiety and the extreme concern about STIs and HIV can still damage relationships and hinder healing. As one man explained:

*“Although you did not repudiate your wife, your heart is twisted (Kahele).”*

This kind of partial acceptance can have long-term negative consequences for both the survivor and her family. Men described how they are still the target of malicious gossip, and may be called stupid or naïve for his choice to accept. Survivors sense this resentment and may live a fractured life where she is only going through the motions of being in a family or marriage. As one man stated: *“Sometimes she will be excluded from normal married life (Kabare).”*

Eventually, she may experience such poor treatment that she chooses to leave her family out of desperation. Once man stated:

*“One can keep her, but the poverty of the family may mean that the family rejects the raped woman, or one can keep her but because of the lack of necessary means, the family cannot meet the needs of the raped woman. The raped woman, herself, leaves the house to search for ways to support herself (Kabere).”*

Another indicated:

*“Families who have no means may well keep the raped woman, but will not be able to seek treatment or after the incident she will not find a man to take her in marriage. After staying a long time in the family, she decides herself to leave to misbehave or prostitute herself, that which may cause her to die of different diseases because neither she nor the family knew how to manage (Katana).”*

## Discussion

The focus group discussions with male relatives of survivors of sexual violence in this study highlight the complex interplay of factors that influence whether a man ultimately decides to reject, accept, or partially accept a female relative that has experienced sexual violence.

Blood relatives of survivors appear to face different pressures than husbands in the aftermath of sexual violence. For husbands or spouses in this study, the added dimension of sexuality – including the pressure to bear and raise children as a couple and the fear of HIV and STIs – contributed to considerations around stigma, acceptance and paths to healing as a couple. While most men recognised that sexual violence was, at its core, a non-consensual act, husbands of survivors felt that once a woman had been with another man, regardless of whether it was forced, the marriage was voided. In some discussions, men still seemed to blame victims of violence even while noting that there was clear use of force during an attack. These contradictory and complex perceptions of rape, including not knowing where to attribute feelings of guilt and shame, played into men’s struggle to know how to respond after sexual violence, and corroborate other existing research in the field [14, 15].

A husband’s impulse to dissolve a marriage was compounded by intense social stigma directed against men themselves as well as toward the survivor. Participants described how they were labelled as ‘weak’ and ‘useless’ by members of their community. Influence from close family members (for instance the husband’s mother or siblings), coupled with wider societal pressure often resulted in a man choosing to reject his wife, even if it was not his first instinct. Gender norms and household roles, particularly regarding women as mothers and caretakers, could strongly influence a man’s decision on how to respond to rape. Kohli and colleagues [14] also described the difficult balance that men faced of wanting to accept a relative that had been raped, yet feeling compelled to reject her based on societal norms. It is not clear whether the level of status of a man in his community impacts his decision to accept or reject a woman, but in line with other previous research [2], men appear to feel compelled to conform with – often potentially harmful – cultural norms.

The fear of contracting sexually transmitted infections, and particularly HIV, was one of the most important factors influencing rejection when the male head of house was the husband. Since sexual relations are a perceived fundamental part of marriage, men felt that if a woman had potentially been infected with a STI, a future relationship was impossible. Respondents also felt strongly that rebel forces had tried to spread HIV as a way of destroying a community bonds and causing dishonour among families. The fear of HIV was pervasive to the point that almost all survivors were assumed to be infected and any husband who chose to remain with his wife after an attack was labelled a “walking dead man”. This finding is consistent with existing literature that addresses militarised rape, where the general consensus is that sexual violence is acknowledged as a means of controlling communities [16]. The compounded challenges of lost livelihood, a survivor’s potentially diminished economic contribution, and the added burden of medical fees push family members choose to reject women who are most vulnerable and in need of support.

Pathways to acceptance were described less frequently in focus groups – often the ultimate decision to support a survivor was based on an accumulation of interacting factors. However, a number of factors did promote family unity. People of influence, for instance religious leaders in the community, had the capacity to encourage men to integrate a survivor into their home; in some cases, men were encouraged by local leaders to accept and support survivors. This was perceived as a force for social cohesion when messages were consistent and delivered by a respected figure. In cases of married couples, a survivor might be allowed to stay in the house if there were young children who would need caring for. However, it is not obvious whether acceptance occurs because of concern for the children in the household, or because caring for children is seen as incompatible with a man’s role. It is important to note that, even though a man may allow a survivor to fulfil her role as a mother, she might still be marginalised or stigmatised within the home and no longer be perceived as a wife or sexual partner.

As described in a previous study [5], women who could continue to contribute financially were more readily accepted into the household, with results of this study demonstrating similar behaviour. Conversely, if a survivor could no longer work or required costly medical treatment, she would be much more likely to be rejected. Importantly however, men in the focus groups discussed how genuine love and affection could encourage a man to withstand social and financial pressures to accept a relative after sexual violence. Focus group respondents noted that faith communities in general encourage comfort and kindness towards female sexual violence survivors. Yet, some leaders and places of worship advocate strongly for the rejection of survivors from the community and their homes. Such advocacy – whether promoting rejection or acceptance – was described as a key influencing factor for men in their decision-making.

Male relatives of survivors are often very difficult to reach and may be reluctant to speak about a topic as sensitive as sexual violence against a female family member. These data provide insight into men’s experiences in dealing with SGBV in their household. This study is one of very few efforts to access both men who continued living with female survivors and those who have chosen to reject. The latter population is considerably harder to identify and more challenging to access. However, these represent some of the most important narratives when trying to understand the problem of stigmatisation of victims of sexual violence, and subsequent outcomes. Often spouses and other close male family members can play important roles in providing immediate care and support, both physically and mentally, in the aftermath of sexual violence, and these efforts should be encouraged, alongside the more complex decision between acceptance and rejection. It is also essential to note that factors influencing acceptance may not necessarily lead to full reintegration of the survivor into the home, or indeed outweigh the factors driving men to reject a woman. For instance, husbands described how their families felt unstable and the marriage had “deteriorated” even if a survivor was accepted in the home. Being rejected or abandoned at one point did not necessarily mean a survivor could never be accepted in the future. Conversely, being accepted did not mean a survivor could not simultaneously feel shamed and ostracised by other family and community members. These findings emphasise how acceptance and rejection sit upon a fluid spectrum that survivors and their families navigate over time.

### Limitations

The dynamics of the focus group discussions may have been influenced by the presence of a foreign researcher, and the fact that some of the subject matter being discussed was sensitive. The research team attempted to mitigate these effects by having focus group moderators with deep experience working on issues related to stigma. In focus groups, there is also a risk that more dominant personalities may influence the conversation and that less well-accepted or non-conventional views do not emerge. To address this issue, focus group moderators were trained to elicit feedback from all participants and to encourage debate within the discussions. Recruiting through a service-providing organisation may have impacted the sample representativeness. Men who hold the most strongly stigmatising views might be those least likely to be identified through a service provision organisation. Focus group moderators attempted address this challenge by asking men to discuss community norms and practices in general rather than dwell on personal experience. An emphasis was placed on understanding both accepting and stigmatising norms towards survivors.

### Recommendations

This work provides insight into concrete ways to improve programming for survivors of sexual violence and their families. Firstly, it emphasises the need for holistic responses to sexual violence. Programmes that address victims’ mental health needs without taking into account their social support structures provide only a partial response. Ideally, programmes would deliver not only one-on-one counselling for women, but also for key members of their household. Family mediation sessions are also reported to be quite effective by local NGOs. This research also stresses the potential role that influential figures in the community can play, in helping to reduce stigma. Customary leaders, religious figures, and respected community members can all play an important role in setting positive examples and encouraging social inclusion. Finally, these results emphasise the extent to which men themselves are targets of gossip, rejection and isolation if a woman in their household has experienced rape. This targeted stigmatisation of men has not been directly addressed in community sensitisation campaigns the authors are aware of. While many organisations rightly address the mental health needs of survivors, a very limited number also provide psychosocial treatment for men. In previous research, women have stated that social rejection after rape can be as traumatic as the original attack, and can hold life-long consequences [17]. Men described their own trauma, but may not have fully recognised the role it played in their treatment of female relatives. For this reason, directly addressing the trauma of those who have the greatest influence over rejection – male family members – can be a powerful investment not only to help male relatives, but survivors as well. As Barker [3] points out, empowerment of women will only occur when men are fully engaged as advocates and change agents. Future research should further explore social attitudes related to male sexual violence survivors – in many cases, this type of violence is viewed as even more sensitive and merits further research.

## Conclusion

This study provides a unique look at the experiences of sexual violence from the perspective of male relatives. Few previous studies have explored the factors that influence men’s responses to sexual violence perpetrated against a female family member. Two overarching strands of dialogue arose from the focus groups: factors driving rejection and pathways to acceptance. Drivers of rejection included the fear of sexually transmitted infections, the personal stigmatisation and social isolation of men and the financial or logistical inability to provide adequate healthcare for survivors as drivers of rejection. Love and affection, financial contributions by the survivors and children in the home were described as pathways to acceptance. It is particularly important to understand drivers of acceptance, because female survivors of sexual violence in DRC state that the reaction of a male relative after rape can be the most pivotal factor in promoting or impeding recovery. Ultimately, men described a complex spectrum of responses to survivors of violence, from full rejection to full acceptance. Where a man fell on this spectrum was influenced by a complex set of social and psychological factors. Encouragingly, men noted that services, counselling and advice from respected figures could help them move along they could move along the continuum from rejection and isolation of female relatives, to a place of understanding and acceptance. This study provides a strong methodological basis for future research, to explore the factors raised here, or extend to populations elsewhere in DRC and beyond.

## Additional file

Additional file 1: Topic Guide. Topic Guide: Men with Relatives Affected by Sexual Violence. Questionnaire to begin after consent script is read and verbal informed consent is obtained from each participant. (DOCX 16 kb)

## Abbreviations

AIDS: Acquired immunodeficiency syndrome
CAMPS: Centre d’assistance médico psychosociale
DRC: Democratic Republic of Congo
HIV: Human immunodeficiency virus
NGO: Non-governmental organisation
STI: Sexually transmitted infection
WHO: World Health Organization

## Footnotes

1 According to the World Health Organization [6], sexual violence is defined as “any sexual act directed against a person’s sexuality using coercion, by any person regardless of their relationship to the victim” (page 149). This terminology includes *rape*, as well as a range of other sexually violent acts, across many settings. This article will reflect the terminology utilised by participants, and also to accurately refer to the act in question. Thus, both *rape* and *sexual violence* are used throughout.

2 The use of the word *victim* in this article reflects the language used by focus group participants, and is not meant to deprive sexual violence survivors of their agency or strength. Similarly, the use of *survivor* is not meant to overlook the mistreatment beyond the control of those who are victimized. The word “victim” is used more generally when referring to women who experienced sexual violence, and it is unclear whether they survived the experience.
