# Supplementary material for: Rejection, acceptance and the spectrum between: understanding male attitudes and experiences towards conflict-related sexual violence in eastern Democratic Republic of Congo
Source: BMC Womens Health. 2017 Dec 8;17:127. doi: 10.1186/s12905-017-0479-7 (PMC5721471; doi:10.1186/s12905-017-0479-7)
Supplement: Additional file 1: — Topic Guide. Topic Guide: Men with Relatives Affected by Sexual Violence. Questionnaire to begin after consent script is read and verbal informed consent is obtained from each participant. (DOCX 16 kb) [file 12905_2017_479_MOESM1_ESM.docx]

**Topic Guide**

**Men with Relatives Affected by Sexual Violence**

Questionnaire to be read after consent script is read and consent is obtained from each participant

*Introduction*

1. How do men get information about what’s happening in your community?
2. Who is responsible for making decisions in your community? In your household?
3. Who do men go to for help when they have problems?
4. Here in Congo, people talk about sexual violence in many different contexts. What do you understand by the phrase sexual violence?
5. When did sexual violence become a problem in your community?
6. Has the problem of sexual violence gotten better, worse or stayed the same since the start of the war?
7. What types of sexual violence do women here face?

*Community and Men’s Reactions to Sexual Violence*

1. Here in Congo, men can also be traumatized by sexual violence against women. Could you tell me about this?
2. How do husbands cope with sexual violence against their wives?
3. How do men cope with sexual violence against their sisters/daughters/mothers?
4. There are times when men choose to not to have his wife stay in the home after rape. Could you tell me about this?
5. What are some of the reasons a man might not have his wife stay in the home after rape?
6. Even when a husband and wife stay together after rape, the marriage can change. Could you tell me about this?
   - - - Prompt for how his attitudes/feelings may have changed
       - How does a couple’s daily activities change?
       - How does a couple’s intimacy change?
7. What are some of the reasons a man might accept his wife and allow her to stay in the home after rape?
8. If a woman has a child born of rape, how does this affect the husband and the marriage?
9. If a woman has a physical problem as a result of rape, how does this affect the husband and the marriage?
10. If a woman has emotional problems as a result of rape, how does this affect the husband and the marriage?
11. If the husband thinks his wife might have an infection as a result of rape, how does this affect the husband and the marriage?
12. How does the economic situation in the household affect the way a husband might treat his wife after rape?
13. If there are children in the household, how might this affect the way a husband might treat his wife after rape?
14. If men or other family members witnessed the sexual violence, how might this affect the way a husband treat his wife after rape?

*Influences on decision making*

1. How do local customs influence how men treat women who have been raped?
2. How do community leaders influence how men treat women who have been raped?
3. How do a man’s parents influence how he treats his wife after rape?
4. How do a man’s other relatives influence how a he treats his wife after rape?
5. How do a man’s friends influence him and how he treats his wife after rape?
6. What changes would you most like to see in your community with respect to survivors of sexual violence?
7. What changes would you most like to see in your community with respect to families of survivors of sexual violence?
8. What kinds of programs would you like to see in your community that are not currently offered there?
9. Are there any other issues you would like to bring up?
